# Comparative Proteomic Analysis of *Aedes aegypti* Larval Midgut after Intoxication with Cry11Aa Toxin from *Bacillus thuringiensis*

**DOI:** 10.1371/journal.pone.0037034

**Published:** 2012-05-16

**Authors:** Angeles Cancino-Rodezno, Luis Lozano, Cris Oppert, Julieta I. Castro, Humberto Lanz-Mendoza, Sergio Encarnación, Amy E. Evans, Sarjeet S. Gill, Mario Soberón, Juan L. Jurat-Fuentes, Alejandra Bravo

**Affiliations:** 1 Departamento de Microbiología Molecular, Instituto de Biotecnología, Universidad Nacional Autónoma de México, Cuernavaca, Morelos, Mexico; 2 Programa de Genómica Evolutiva, Centro de Ciencias Genómicas, Universidad Nacional Autónoma de México, Cuernavaca, Morelos, Mexico; 3 Department of Entomology and Plant Pathology, University of Tennessee, Knoxville, Tennessee, United States of America; 4 Unidad de Proteómica, Centro de Investigación Sobre Enfermedades Infecciosas, Instituto Nacional de Salud Pública, Cuernavaca, Morelos, Mexico; 5 Department of Cell Biology and Neuroscience, University of California Riverside, Riverside, California, United States of America; University of Crete, Greece

## Abstract

Cry toxins produced by *Bacillus thuringiensis* bacteria are environmentally safe alternatives to control insect pests. They are pore-forming toxins that specifically affect cell permeability and cellular integrity of insect-midgut cells. In this work we analyzed the defensive response of *Aedes aegypti* larva to Cry11Aa toxin intoxication by proteomic and functional genomic analyses. Two dimensional differential in-gel electrophoresis (2D-DIGE) was utilized to analyze proteomic differences among *A. aegypti* larvae intoxicated with different doses of Cry11Aa toxin compared to a buffer treatment. Spots with significant differential expression (p<0.05) were then identified by liquid chromatography-tandem mass spectrometry (LC-MS/MS), revealing 18 up-regulated and seven down-regulated proteins. The most abundant subcategories of differentially expressed proteins were proteins involved in protein turnover and folding, energy production, and cytoskeleton maintenance. We selected three candidate proteins based on their differential expression as representatives of the different functional categories to perform gene silencing by RNA interference and analyze their functional role. The heat shock protein HSP90 was selected from the proteins involved in protein turnover and chaperones; actin, was selected as representative of the cytoskeleton protein group, and ATP synthase subunit beta was selected from the group of proteins involved in energy production. When we affected the expression of ATP synthase subunit beta and actin by silencing with RNAi the larvae became hypersensitive to toxin action. In addition, we found that mosquito larvae displayed a resistant phenotype when the heat shock protein was silenced. These results provide insight into the molecular components influencing the defense to Cry toxin intoxication and facilitate further studies on the roles of identified genes.

## Introduction

Insecticidal crystal toxins (Cry) are pore-forming toxins (PFT) produced by *Bacillus thuringiensis* (Bt) bacteria as crystalline inclusions during the sporulation phase of growth [1]. The Cry toxins are highly specific against different insect orders such as Lepidoptera, Diptera, Coleoptera, or Hymenoptera, as well as to nematodes. These proteins are harmless to humans and biodegradable, and are thus considered environmentally safe alternatives to control insect pests in agriculture and insects that are vectors of human diseases. The Cry proteins show a complex mechanism of action involving multiple and sequential binding interactions with specific protein receptors located in the microvilli of midgut epithelial cells. The interaction with these receptors depends on a change in the oligomeric state of the toxin, from monomeric to oligomeric, leading finally to insertion of the oligomeric form of Cry toxin into the membrane, forming lytic pores that causes cell swelling, lysis and insect death [2], [3].

Many other PFT are produced by different pathogenic bacteria that also kill their targets by making pores in the cell membrane of their target cells, affecting cell permeability and disrupting cellular integrity [4]. Eukaryotic cells have evolved different defense responses to cope with these virulent factors. The innate immune system plays an important role to protect cells from PFT, and it was shown that the MAPK p38 and JNK pathways activate survival responses in several mammalian cell types after treatment with different PFT such as aerolysin, pneumolysin, streptolysin O, α-hemolysin, and anthrolysin O [5]. Recently, efforts to understand the global responses that eukaryotic cells use to overcome the action of different PFT have been documented. Studies of the *Caenorhabditis elegans* response to Cry5 toxin, such as microarrays and a genome-wide RNA interference (RNAi) analysis, showed that the *C. elegans* response is quite complex since 0.5% of the genome of this animal participates in the protection from PFT attack, with MAPK and JNK having pivotal roles in activating transcriptional and functional responses [6], [7].

In insects, the genomic response to insecticidal Cry toxins is poorly understood. It was shown that MAPK p38 pathway is activated after Cry-toxin intoxication in two insect orders, Lepidoptera and Diptera [8]. Silencing of p38 by RNAi caused larvae to be hypersensitive to toxin action, demonstrating that the MAPK p38 pathway plays a protective role *in vivo* against Cry toxins action in both insect orders [8].

Recent reports characterized some of the defensive response of insects to Cry toxin intoxication. These include a proteomic analysis in *Helicoverpa armigera* after ingestion of Cry1Ac [9], and the analysis of subtraction hybridization libraries in *Choristoneura fumiferana* larvae treated with Cry1Ab toxin [10], [11]. Both studies used 4^th^ or 5^th^ instar larvae exposed to sublethal toxin concentrations [9], [10], [11]. None of these studies analyzed the functional role of the proteins that were identified as participants in the insect response to Cry toxin intoxication.

In this work, we analyzed the proteomic response of *Aedes aegypti* mosquito larvae after intoxication with two different doses of Cry11Aa toxin, medium lethal concentration (LC_50_) and a lethal concentration that kills 10% of the larvae (LC_10_). Most midgut proteome alterations were observed at the LC_50_ suggesting that a defensive response was triggered. dsRNA-mediated silencing was then used to analyze the functional role of selected proteins whose expression was altered after Cry11Aa toxin exposure. These functional experiments identified two proteins as involved in defense-response since larvae affected in their expression were hypersensitive to toxin action. Silencing of a third protein resulted in a resistant phenotype, suggesting that this protein is involved in successful larval intoxication.

## Results

### Proteomic profile of *Aedes aegypti* after intoxication with Cry11Aa toxin

**Figure 1:**
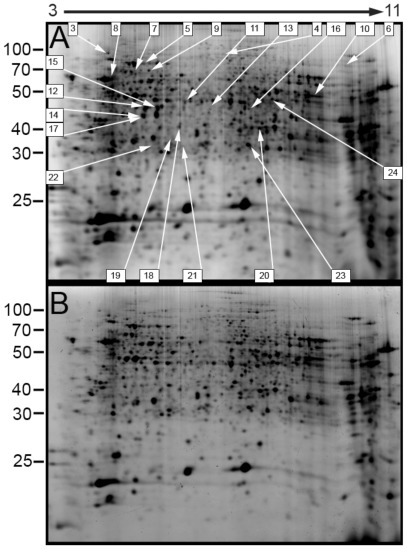
Representative 2D SDS-PAGE protein spot maps of larval midgut proteomes from larvae treated with buffer (A) or an LC_50_ dose of Cry11Aa (B). Spots selected for identification by nano LC/MS/MS are indicated and numbered as in Table 1. Spot 18 corresponds to spots 18A and 18B. The gel pH gradient is denoted above the figure. Estimated protein molecular weights are shown at the left of the figure in kilodaltons.

Through bioassays we determine that the lethal concentration of Cry11A that kills 10% of the larvae after 24 h (LC_10_) was 6.8 ng toxin/ml (2.1–18.5 confidence interval), while the LC_50_ value was 154 ng toxin/ml (94.5–322.0 confidence interval). We fed Cry11A toxin to *A. aegypti* larvae for 5 h with these two toxin concentrations, and then performed a 2-dimensional differential in-gel proteomic (2D-DIGE) analysis to characterize the insect response under these conditions. As control, we used larvae treated with a corresponding volume of toxin buffer. Two protein extracts labeled with Cy3 and Cy5 probes were loaded in each gel in addition to an internal standard labeled with a Cy2 probe, allowing normalization of abundance ratios to provide multivariable experiments with great statistical power. When using the LC_10_ Cry11A dose, we only identified two proteins as differentially present in Cry11A compared to control sample, the F_0_F_1_-type ATP synthase subunit beta and a serine-type endopeptidase (Table 1). In contrast, we detected a total of 22 protein-spots that were differentially expressed in response to treatment with LC_50_ dose of Cry11Aa compared to control (Fig. 1, Table 1). The identity of these proteins was determined by trypsin digestion and analyzed by nano liquid chromatography followed by tandem mass spectrometry (LC-MS/MS). Protein data were searched against the concatenated forward and reverse Uniprot *A. aegypti* database. Out of the 22 proteins differentially detected in the LC_50_ treatment 21 of them were annotated (Table 1). An identified uncharacterized protein matched with 83% sequence identity to apolipophorin from the mosquito *Culex quinquefasciatus* in BLASTp searches of the NCBInr database.

**Table 1:** Identification of proteins with significantly altered levels in *A. aegypti* larvae treated with an LC_10_ (gray cells) or an LC_50_ (white cells) dose of Cry11Aa toxin compared to buffer controls.

Spot number	Top match^a^	Accession number^b^	Percent coverage^c^	Unique/total spectra	KOG^d^	Protein levels^e^	Previous reports
1	F0F1-type ATP synthase beta subunit	Q17FL3	20%	8/15	C	+1.15	9, 23, 25
2	Serine protease	Q16ZF3	39%	10/26	O	−1.03	
3	Heat shock protein	Q16FA5	46%	67/93	O	−2.15	9, 10, 26
4	Eukaryotic translation elongation factor	Q0IFN2	11%	9/11	J	+2.24	
5	V-type proton ATPase catalytic subunit A	O16109	45%	33/62	C	−2.00	
6	Putative uncharacterized protein^f^	Q16UB8	14%	46/46		+1.44	
7	V-type proton ATPase catalytic subunit A	O16109	59%	50/178	C	+2.11	
8	Spectrin	Q16EQ1	14%	34/34	Z	+1.3	
9	V-type proton ATPase catalytic subunit A	O16109	33%	23/49	C	+2.36	
10	Aspartate ammonia lyase	Q16ZL0	36%	18/23	C	+2.00	
11	Actin	Q178A9	11%	3/4	Z	−1.93	9, 24, 26, 27
12	Actin	Q178A9	58%	29/44	Z	−3.32	9, 24, 26, 27
13	Estradiol 17 beta-dehydrogenase	Q173X5	35%	33/47	I	+2.86	
14	Actin	Q17KG3	51%	5/11	Z	−2.02	9, 24, 26, 27
15	Actin	Q16QR7	33%	4/35	Z	−4.35	9, 24, 26, 27
16	Alcohol dehydrogenase	Q176A3	17%	8/16	Q	+1.84	
17	3-hydroxyacyl-CoA dehydrogenase	Q0IEU5	26%	7/7	I	+4.89	
18a	Serine protease inhibitor 4	Q0IEW2	16%	5/5	V	+6.53	10
18b	Actin	Q178A9	17%	5/5	Z	+6.53	9, 24, 26, 27
19	Vacuolar ATP synthase subunit e	Q1HQT6	62%	26/40	C	+2.55	9, 23, 25, 26
20	Arginine or creatine kinase	Q1HR67	72%	74/214	C	+2.14	9
21	ATP synthase subunit beta vacuolar	Q9XYC8	13%	6/9	C	+1.51	9, 23, 25
22	Peroxiredoxin 6	Q17IM5	35%	7/7	O	+2.41	
23	Triosephosphate isomerase	Q17HW3	53%	15/15	I	+2.10	
24	Actin	Q178A9	11%	3/4	Z	+1.49	9, 24, 26, 27

a All matches were to sequences from *Aedes aegypti*.

b UniProtKB/Swiss-Prot *A. aegypti* database.

c Defined as the percentage of all the amino acids in a protein that were identified from sample spectra.

d C, Energy production and conversion; I, Lipid transport and metabolism; J. Translation, ribosomal structure and biogenesis; O. Post-translational modification, protein turnover, chaperones; Q, Secondary metabolites biosynthesis, transport and catabolism; V, Defense mechanisms; Z, Cytoskeleton.

e Fold difference in larvae treated with Cry11Aa toxin compared with control (buffer) treatment.

f BLASTp searches of the NCBInr database with this uncharacterized protein returned high identity (83%) matches to *Culex quinquefasciatus* apolipophorin (XP_001849310).

**Figure 2:**
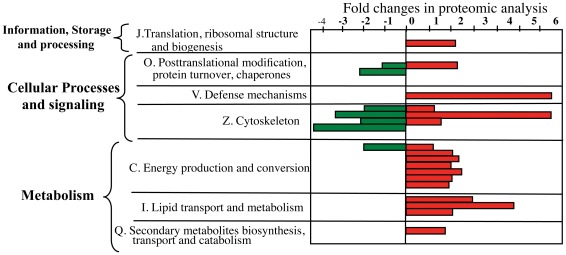
Representation of the functional annotation analysis for KOG of all genes that showed 0.5 fold higher or lower change in protein expression.

Each of the differentially expressed proteins was identified in the VectorBase database and analyzed to determine the functional category of the corresponding genes using the eukaryotic orthologous groups (KOGs) of the Cluster of Orthologous Groups (COG) database. The functional annotation analysis for KOG of all genes that showed 0.5 fold higher or lower change in protein expression are summarized in Figure 2, where sequences corresponding to the “Information storage and processing” group fell into one subcategory (named J), the “cellular processes and signaling” group into three subcategories (named O, V and Z, see Fig. 2 and Table 1), and “metabolism” into three subcategories (named C, I, and Q, see Fig. 2 and Table 1). The most abundant subcategories of differentially expressed proteins were proteins involved in posttranslational modification, protein turnover and chaperones (subcategory O), cytoskeleton (subcategory Z), energy production and conversion (subcategory C) and lipid transport and metabolism (subcategory I) (Fig. 2, Table 1). In addition, we found a single protein in subcategories J (translation, ribosomal structure and biogenesis), Q (secondary metabolite biosymthesis, transport and catabolism), and V (defense mechanisms). Among the proteins involved in energy production and conversion we found two proteins directly involved in ATP synthesis such as the ATP synthase subunit beta and the Vacuolar ATP synthase subunit epsilon. Other proteins in this subcategory were involved in energy production by catalysis, including arginine/creatine kinase, aspartate ammonia lyase, and the catalytic A subunit of the V-ATPase. The proteins identified that are involved in cytoskeleton were actin and spectrin. Three actin forms were identified, and while two of them were down-regulated, the third form (Q178A9) was detected two times as down-regulated (spots 11 and 12) and two times as up-regulated (spot 18b and 24). Interestingly, these protein spots were localized to diverse isoelectric points, suggesting that they may represent different post-translationally modified forms of this protein, and that Cry11Aa intoxication increases the levels of a specific isoform. Among the proteins involved in posttranslational modification, protein turnover and chaperones we found a protein with chaperone function, the heat shock protein HSP90, and a protein involved in antioxidant activity, peroxiredoxin 6. In the group of proteins involved in lipid transport and metabolism we identify estradiol 17 beta-dehydrogenase, 3-hydroxyacyl-CoA dehyrogenase, 3-hydroxyacyl-CoA dehyrogenase and triosephosphate isomerase. Finally, we also found a translation elongation factor, alcohol dehydrogenase, and serine protease inhibitor-4 (serpin), as representatives of the J, Q, and V subcategories, respectively.

In order to identify the biological pathways that were activated after toxin ingestion, we mapped the differential expressed proteins to canonical signaling pathways found in the Kyoto Encyclopedia of Genes and Genomes (KEGG). The KEGG analysis showed the immune system NOD-like receptor pathway since the heat shock protein HSP90 participates in this pathway. In addition some proteins of the pathways of glycolysis, citrate (TCA) cycle and fatty acid metabolism pathways were also activated by Cry11Aa treatment, suggesting activation of carbohydrate and lipid metabolism after toxin intoxication.

### Functional studies of selected proteins by RNA interference analysis

**Figure 3:**
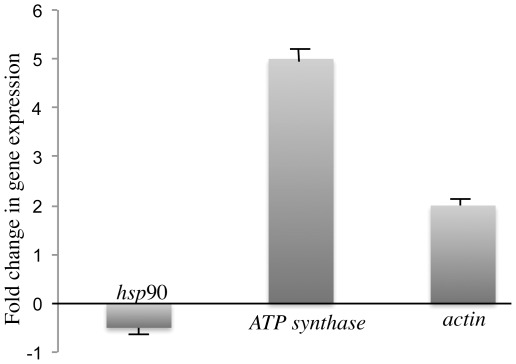
Regulation of *actin, hsp*90 and *ATP synthase* genes in *Aedes aegypti* larvae after 5 h intoxication with LC_50_ of Cry11Aa toxin analyzed by quantitative qRT-PCR assays.

**Figure 4:**
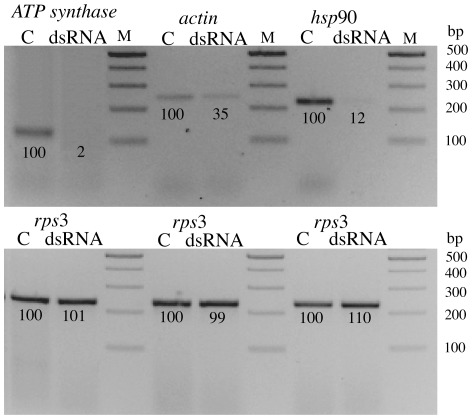
Silencing of actin, hsp90 and ATP synthase by RNAi in *Aedes aegypti* larvae. The expression of these proteins was silenced by feeding dsRNA to *A. aegypti* larvae. The expression of each gene was analyzed by RT-PCR assays. Numbers under the bands are percentage in relation to the control band, after densitometry analysis using ImageJ program. The control bands correspond to non-silenced larvae, which were labeled with a C and were considered as 100%. M, molecular size marker in bp.

To understand the role of some proteins in the response to Cry toxins, we selected three proteins to perform functional studies by RNAi. Candidate proteins, chaperone HSP90, actin and ATP synthase subunit beta were selected based on their differential expression as representatives of a different functional category. To validate the proteomic data we performed quantitative real-time (qRT) PCR analyses using isolated RNA from independent experiments, with and without Cry11Aa toxin, comparing larvae treated with an LC_50_ for Cry11A to buffer controls (Fig. 3). The qRT-PCR results validated the proteomic results for HSP90 and ATP synthase. In the case of actin, we measured transcript levels for the form that was found to display diverse regulation in response to toxin (protein Q178A9). We detected that the corresponding gene (AAEL005961) was up-regulated in larvae treated with Cry11A (Fig. 3). The molecular chaperone HSP90 was selected from the proteins involved in posttranslational modification, protein turnover and chaperones since this protein is involved in response to stress through protein folding and cell signaling [12]. Contrary to up-regulation detected under stress, in our proteomic analyses HSP90 was repressed and it was also repressed in the transcriptomic studies performed with *Ch. fumiferana* larvae after feeding with Cry1Ab toxin [10]. Successful silencing of HSP90 was confirmed by RT-PCR with decreased transcript levels when compared to untreated larvae (Fig. 4). In contrast, there was no change in *rps3* gene expression, which was used as an internal control. The effect of silencing the HSP90 protein on Cry toxin susceptibility showed that larvae became highly tolerant to Cry11Aa toxin, showing an LC_50_ value four-fold higher than the non-silenced larvae (Table 2). Silencing of HSP90 did not affect larval development up to 4^th^ instar or mortality of silenced and not-silenced larvae in the control condition, without toxin addition, suggesting no-major effects on larval viability.

**Table 2:** Susceptibility to Cry11Aa toxin intoxication after silencing the protein expression of selected targets by RNAi.

Gene silenced	Gene accession	Protein accession	Activation or repression	Cry11Aa LC_50_ ng/ml (95% confidence interval)	Phenotype
None	-	-	-	154 (64.5–322.0)	none
Heat shock protein	AAEL011704	Q16FA5	−2.15	650 (381.3–1290.1)	4 fold tolerant
ATP synthase beta subunit	AAEL003393	Q17FL3	+1.5	33 (12.3–60.4)	4 fold hypersensitive
Actin	AAEL005961	Q178A9	+1.49	65 (28.8–120.2)	2 fold hypersensitive

For a protein involved in energy production we selected to silence the ATP synthase subunit beta, which is directly involved in ATP synthesis coupled to proton transport, forming part of the F_1_ complex of ATPase. This protein was selected because its expression increased in our proteomic studies at the LC_10_ toxin dose and previous reports using *H. armigera* larvae fed with Cry1Ac [9] or *Ch. fumiferana* larvae after Cry1Ab ingestion [10] also showed elevated levels of this protein. The functional role of this protein in the defense response to Cry toxin action was never analyzed, and that is the reason why we selected to silence this protein by RNAi. However, resistant larvae of *Plodia interpunctella* also showed higher levels of F_1_F_0_-ATPase [13], suggesting that higher levels of this protein may help in protection to toxin damage. Silencing of this ATP synthase subunit by RNAi resulted in lower transcript levels, as shown in the RT-PCR analysis (Fig. 4). In bioassays, silenced larvae became hypersensitive to Cry11Aa toxin action showing an LC_50_ that was four times lower than the control larvae (Table 2). The silenced larvae grew slightly slower than control larvae, since they required three weeks to reach the 4^th^ instar in contrast to control larva that developed into 4^th^ instar in two weeks. However, once they reached the 4^th^ instar they looked healthy under control conditions without toxin intoxication and had similar size as the control larvae. One advantage of RNAi vs. gene knockout is that in RNAi the expression of silenced protein is reduced in the cell and not completely eliminated, this could explain why silencing a protein that may play an important role in cell viability could result in non-lethal phenotypes.

As representative of the cytoskeleton protein group we silenced the actin gene that corresponds to actin protein Q178A9, since this actin form was detected two times down-regulated (spots 11 and 12) and two times up-regulated (spots 18B and 24). Also because the expression of this gene was found to be over-expressed in Cry11Aa-treated larvae by qRT-PCR. Actin was identified to be up-regulated in the proteomic studies performed in *H. armigera* larvae fed with Cry1Ac toxin [9]. In addition, actin was identified as a Cry1Ac binding proteins in Lepidoptera [14]–[16] and as a Cry4Ba binding protein in *A. aegypti* brush border preparations [17]. Silencing actin expression by RNAi was not complete since lower transcript levels were observed in the RT-PCR analysis (Fig. 4). However, the insecticidal activity of Cry11Aa spore-crystal suspension in *actin*-silenced and in control larvae showed a two fold decrease in the LC_50_ of silenced compared to control larvae, suggesting increased sensitivity to the toxin (Table 2). The actin-silenced larvae also developed slowly requiring three weeks to reach the 4^th^ instar. However the size of the larvae at the end of the development was similar to the non-silenced larvae and they looked healthy under control conditions without toxin intoxication.

## Discussion

The systematic analysis of how insects respond to Cry toxins is likely to provide new tools for improving insecticidal activities against certain pests and to cope with the potential risk of resistance evolution to these toxins. The transcriptional response of *Ch. fumiferana* to sublethal doses of Cry1Ab was previously analyzed [10]. That study identified 156 clones from a cDNA subtractive library as differentially expressed after exposure to Cry1Ab. Most of these clones were predicted to be involved in catalytic activity, binding, or structural function. However, altered transcriptional patterns were observed in only a few proteins by real time-PCR showing that serine protease was enhanced as well as cytochrome P450, while a metalloprotease and a heath shock protein were repressed [10]. In an alternative report, the proteomic profile of *H. armigera* brush border membrane proteins after Cry1Ac toxin treatment was analyzed [9]. The authors identified some proteins that showed higher abundance after toxin ingestion, such as aminopeptidase N, V-ATPase subunits, and actin. In contrast, a trypsin-like protease was reported to decrease in response to intoxication [9]. However, the functional role of the identified genes and proteins on the insect response to Cry toxin action was not analyzed, which could yield more information on their participation in the larval defense response.

In this work the proteomic profiles of *A. aegypti* larvae intoxicated with two different doses of Cry11Aa toxin compared to buffer-treated larvae were analyzed. First, we decided to analyze a moderate Cry11Aa dose (LC_10_) to assure an active response of the insect gut cells in conditions where the gut epithelium recovers from toxin damage. Nevertheless, the analysis of this moderate response allowed us to identify only two proteins with significant altered expression levels, the F_0_F_1_-type ATP synthase beta subunit and a serine-type endopeptidase. These results prompted us to analyze a higher toxin dose (LC_50_) using incubation times that did not revealed significant tissue damage in microscopic histological inspection. The proteomic analysis of the LC_50_ treatment showed an active response of the insect gut cells as revealed by the identification of 22 protein-spots with significant changes in their expression levels. Identification of these proteins by mass spectrophotometry revealed that the most abundant subcategories of proteins identified were proteins involved in protein turnover and folding, energy production, lipid metabolism and cytoskeleton maintenance. These data shows that *A. aegypti* midgut cells respond to Cry11Aa intoxication by activating their metabolism to increase ATP synthesis and lipid biosynthesis and by modifications in cell cytoskeleton and chaperon responses. The activation of lipid metabolism synthesis could be a defensive response to counter membrane damage resulted by Cry11Aa toxin insertion into the membrane and pore formation. Also, up regulation of ATP synthases and V-ATPase suggest that cell intoxicated with Cry toxins respond by increasing their energy profile to counter act toxin action. The V-type proton ATPase is an electrogenic proton pump located in goblet cell apical membranes that couples the energy of ATP hydrolysis to transport protons across the membrane. This protein is important for pH homeostasis, and is responsible for alkanization of the gut lumen and it energizes an electrophoretic K^+^/nH^+^ antiport, playing an important role in midgut ion-transport processes [18]. In a proteomic analysis performed in *H. armigera* larvae intoxicated with Cry1Ac, it was found that a vacuolar ATP synthase subunit B and V-ATPase subunit A were also increased [9], supporting that activation of these enzymes may be a general insect responses to stress conditions such as the Cry toxin action. It is also important to mention that V-ATPase synthase was also identified as Cry1Ac binding protein in *H. virescens* [15] and *H. armigera* [16] and was increased in Cry1Ac-resistant *Plodia interpunctella* [13]. Also, it was found to bind Cry4Ba in larvae of *A. aegypti* [17]. In this work, we silenced one representative protein of this functional group: the ATP synthase subunit beta, which resulted in a fourfold hypersensitive phenotype to Cry11Aa intoxication. The functional participation of ATP synthase on the response to Cry toxin action was not analyzed before and we show here evidence supporting that energy production is necessary to activate defense mechanisms to stress conditions such as Cry toxin action. It is clear that insects affected in ATP synthase would have an effect in fitness costs, with a decreased metabolism activity making them less reactive than healthy-ones to any stress condition, including pore-formation triggered by Cry toxin, showing a hypersensitive phenotype after toxin ingestion.

Regarding to cytoskeleton proteins we found decreased levels of some actin forms, but higher expression of others, as well as increased levels of spectrin after Cry toxin ingestion. Actin is one of the three major components of the cytoskeleton and it is involved in important cellular processes such as cell motility, cell division, vesicle movement, differentiation, and proliferation. Spectrin is the major component of the protein network that covers the cytoplasmic surface of cell membranes linked to short actin filaments. This network is coupled to the membrane bilayer primarily through the association with other proteins such as ankyrin. Cytoskeletal elements interact extensively and intimately with cellular membranes and could promote a cellular response leading to a defense mechanism [19]. Actin was also showed to have an increased expression in the proteomic study performed in *H. armigera* larvae after Cry1Ac ingestion [9] and was previously identified as a putative Cry1Ac binding protein in *Heliothis virescens* [15], *H. armigera* [16] and *Manduca sexta* larvae [14]. Actin was also reported as a binding protein for Cry4Ba in the midgut membrane of the mosquito *A. aegypti* [17]. Since actin forms ordered arrays to support the apical surface of brush border in the midgut it was proposed that contacts between the toxin and actin could occur after insertion the toxin into the membrane. Overall, these data supported that actin and V-ATPase may be playing an important role in toxin mode of action. In this work, we partially silenced the actin gene that we found to be up-regulated. When this actin gene was partially silenced the resulting larvae displayed two-fold higher sensitivity to the Cry11Aa toxin, indicating that this protein may help to overcome Cry11Aa intoxication. It is important to mention that actin is a protein that plays a key role in cellular metabolism, then insects affected in this protein may also have a fitness cost that made them more susceptible to different stress conditions such as the pore forming toxin affecting their midgut cells.

Among the proteins involved in posttranslational modification, protein turnover and chaperones, we found heat shock protein HSP90 as a protein with chaperone function. The chaperone activity of HSP90 is important for stabilizing anti-apoptotic signal transduction pathways, and also has been shown to be important for the biogenesis of certain membrane receptors [12]. In our analysis we found that the HSP90 was down regulated in response to Cry11Aa. When we silenced expression of HSP90 by RNAi we observed a resistant phenotype to Cry11Aa intoxication. These results suggest that HSP90 could participate in a signal transduction pathway involved in cell death in response to Cry11Aa. Alternatively, HSP90 may participate in the assembly of a Cry11Aa receptor molecule in the insect gut. Further work would be necessary to test these hypotheses.

In contrast to previous studies in *C. elegans* on the response to Cry intoxication, our analysis did not reveal any changes in expression of proteins involved in signal transduction, like p38 and JNK pathways [6], [7]. Nevertheless, previous work showed that p38 MAPK is not regulated at the transcriptional and protein level in *A. aegypti* but it was activated by phosphorylation after Cry11Aa toxin exposure [8]. It would be interesting to analyze changes in the phospho-proteome to determine the signal transduction pathways that are actively involved in cell response to Cry11Aa toxin in *A. aegypti*.

Although HSP90, ATP synthase subunit beta, and actin proteins were previously identified in differential analyses of lepidopteran pests after toxin intoxication, their functional role was not demonstrated before. Here we used gene silencing to specifically analyze the participation of these proteins in the response to Cry toxin action and we show that the functions of ATP synthase subunit beta and actin proteins are necessary to have a robust defense response to stress conditions such as Cry toxin intoxication. Also, silencing of HSP90 expression showed that this protein is involved in cell death responses. In addition we identified other proteins that are also modulated after toxin ingestion that may be also implicated in the insect response to toxin action and deserve to be analyzed. The functional role of these other proteins remains to be analyzed. Overall, the results presented here indicate that the response of insect midgut cells to Cry toxin action is complex and involves the modulation of many proteins. Our functional data identify some of these proteins as relevant to the Cry intoxication process. This information represents the basis to understand how insects cope with Cry toxins and could provide tools for improving Cry toxicity to insect pests.

## Materials and Methods

### Bacterial strains and Cry toxin production

Bt strain harboring pCG6-Cry11Aa [20] plasmid was grown at 30°C in nutrient broth sporulation medium with 10 µg/ml erythromycin until complete sporulation. Crystal inclusions were observed under phase contrast microscopy and purified by sucrose gradients [21]. Final crystal samples were suspended in PBS buffer pH 7.4.

### Insect Bioassays and treatments with toxin

Protein concentrations of crystal preparation were determined using the Bradford assay. Bioassays were performed with 4^th^ instar *A. aegypti* larvae, intoxicated for 24-h with different concentrations of Cry11Aa crystal suspensions (0 to 10,000 ng/ml) directly added to 100 ml of H_2_O. Ten 4^th^ instar larvae were used per container and the concentrations causing 10% and 50% mortality (LC_10_ and LC_50_) values were estimated by Probit analysis (Polo-PC LeOra Software).

Intoxication treatments for proteomic analysis were done also with 4^th^ instar *A. aegypti* larvae that were fed for 5 h with LC_10_ and LC_50_ of Cry11Aa crystal suspension. We chose a 5 h treatment time to allow larvae to ingest the toxin without severely affecting their behavior and without causing evident damage to the intestinal tissue at the microscopic histological level. Control larvae were fed with corresponding volumes of toxin buffer.

### Preparation of midgut protein extracts for proteomics analysis

Midguts were dissected from 4^th^ instar *A. aegypti* larvae and pools of 50 entire midguts solubilized in 100 µl of rehydration solution (7 M urea, 2 M thiourea, 4% CHAPS, 40 mM dithiothreitol, 0.5% pharmalyte or IPG buffer [GE Life Sciences], 0.002% bromophenol blue, 2.5 ml) containing protease inhibitors (Complete, Roche Diagnostics) were kept at −80°C until processed. Midgut pools were homogenized with a motorized pellet pestle (Motor Sigma-Aldrich Z359971.1EA) on ice. Midgut protein samples were cleaned using the 2-D Clean-Up Kit (GE Life Sciences) following the manufacturer's instructions. Protein samples were then solubilized in the rehydration solution described above and quantified using the 2-D Quant Kit (GE Life Sciences) as per manufacturer's instructions. Midgut protein sample concentration was adjusted to 50 µg in 125 µl using rehydration buffer. Fifty micrograms of each sample was labeled with 4 pmol/µg protein of Cy3 or Cy5 dye following the manufacturer's instructions (GE Life Sciences). As an internal control, a pooled sample containing 50 µg total protein was labeled with Cy2.

### Differential In-Gel Electrophoresis (DIGE) analysis

Midgut proteomes from control and Cry11A-intoxicated *A. aegypti* larvae were compared using two-dimensional (2D) DIGE to identify proteins with differential expression in response to intoxication. As negative controls we used midgut proteomes from control larvae, without toxin administration. The experimental design included sample randomization and a Cy2-labeled internal standard containing equal amounts of proteins from all of the compared samples. Protein samples labeled with different Cy dyes were randomly combined in sets of two for 2D electrophoresis. A total of four biological replicates, each midgut-extracted protein from a pool of 50 larvae, were used for each treatment. For the first dimension the combined protein samples were used to rehydrate 18 cm immobilized pH gradient (IPG) strips (pH 3 to 11 non-linear, GE Life Sciences) overnight. Each strip also included the Cy2-labeled standard to allow protein spot quantification and comparisons within and between gels. First dimension electrofocusing was run on the IPGphor III (GE Life Sciences) at 20°C with the following settings: step 1, 500 V, 1 h; step 2, 500 V to, 1,000 V, 4 h; step 3, 1000 V to 8,000 V, 3 h, step 4: 8000 V, 1 h. Before the second dimension sodium dodecyl sulfate polyacrylamide gel electrophoresis (SDS-PAGE), the strips were reduced for 10 min with 64.8 mM of dithiothreitol in SDS equilibration buffer (50 mM Tris-HCl [pH 8.8], 6 M urea, 30% glycerol, 2% SDS, 0.002% bromophenol blue), and then alkylated for 15 min with 135.2 mM of iodoacetamide in the same equilibration buffer. The second dimension was carried out in the Ettan DALT Six system (GE Life Sciences). The SDS-PAGE gels used were 15% homogeneous acrylamide gels cast in the laboratory. Electrophoresis was performed using an initial step of 2 W/gel for 25 min followed by 17 W/gel until the dye front reached the bottom of the gel.

Gels were scanned immediately after SDS-PAGE using a Typhoon Imager (GE Life Sciences), optimizing the photomultiplier tubes for each laser to achieve the broadest dynamic range. The proteome maps acquired were loaded on the DeCyder 2D v6.5 software (GE Life Sciences) to analyze protein spot abundance differences between maps. Protein spots were assigned automatically and confirmed using the slope of the signal peaks, area and 3D representations. The spots reported for the LC_50_ treatment all had at least a 1.3-fold significantly different intensity with respect to controls (ANOVA, P<0.05) while in the analysis of the LC_10_ treatment we reduce the cutoff to at least 1-fold to detect proteome differences in respect to controls.

### Protein identification

Protein spots with differential expression in Cry11A-treated larvae compared to buffer controls as determined by the 2D-DIGE analysis were excised using the Ettan Spot picker (GE Life Sciences). Excised protein spots were submitted to NextGen Sciences (Ann Arbor, MI) for identification. Gel plugs were subjected to proteolytic digestion on a ProGest (Genomic Solutions) workstation using bovine trypsin. Formic acid was added to stop the reaction, and the supernatant was analyzed directly using nano liquid chromatography followed by tandem mass spectrometry (LC-MS/MS) with a 30 min gradient on a LTQ Orbitrap XL mass spectrometer (ThermoFisher). Product ion data were searched against the concatenated forward and reverse Uniprot *A. aegypti* database using the Mascot search engine (Matrix Science, London, UK). Search parameters included a fragment ion mass tolerance of 0.50 Da, a parent ion tolerance of 10.0 PPM, and iodoacetamide derivative of cysteine as a fixed modification. Variable modifications considered included S-carbamoylmethylcysteine cyclization (N-terminus), deamidation of asparagine and glutamine, oxidation of methionine, and acetylation of the N-terminus. The database was appended with commonly observed background proteins (cRAP) to prevent false assignment of peptides derived from those proteins. Mascot output files were parsed into the Scaffold 3 (version Scaffold_3_00_07, Proteome Software Inc., Portland, OR) for filtering to assess false discovery rate, which was ≤0.5%, and allow only correct protein identifications. Peptide identifications were accepted if they could be established at greater than 50% probability as specified by the Peptide Prophet algorithm [22]. Protein identifications were accepted if they could be established at greater than 90% probability and contained at least 2 identified peptides. Protein-probabilities were assigned by the Protein Prophet algorithm [23]. Proteins that contained similar peptides and could not be differentiated based on MS/MS analysis alone were grouped to satisfy the principles of parsimony.

### Identification of Orthologous Groups and Metabolic Pathways

The protein sequences of *A. aegypti* proteins identified with the proteomic analyses were obtained from the VectorBase database [24]. The database of orthologous groups for eukaryotic complete genomes (KOG) from the cluster of orthologous groups (COG) database was used to determine the functional category of all identified proteins [25]. All proteins were subjected to a BLASTp search [26] against the KOG database with the *e*-value inclusion threshold set to e^−12^ and an amino acid sequence identity threshold of 30%. Proteins with more than one functional category assignment were excluded. The Kyoto Encyclopedia of Genes and Genomes (KEGG) database [27] links genomic information with higher order functional information. The KEGG Pathway database is a collection of graphical maps representing different cellular processes. KEGG Pathway was used to determine the participation of each protein in one or more pathways by two procedures, a) as in the KOG analysis, all the proteins were subjected to a BLASTp search with the same threshold criteria for the *e*-value and the amino acid identity; b) we used the KEGG Automatic Annotation Server (KAAS) [28] with the BBH (bi-directional best hit) method. Proteins assigned to a pathway by only one method were manually analyzed to determine their participation in that specific pathway.

### RNA Interference (RNAi) assays

Total RNA was isolated from midgut tissue of *A. aegypti* larvae using the RNeasy Kit (Qiagen). One µg of total RNA was used for reverse transcription polymerase chain reaction (RT-PCR) amplification using a First Strand cDNA Synthesis Kit for RT-PCR (AMV, Roche). Specific oligonucleotides (Table 3) were designed using Primer3 Input software (version 0.4.0) [29] to amplify selected genes based on the genome sequence accession numbers corresponding to the identified proteins for ATP synthase beta subunit (AAEL003393), actin (AAEL005961), and heat shock protein (AAEL011704). Amplified cDNA fragments from *A. aegypti* were cloned into a TOPO cloning vector using TOPO TA cloning kit (Invitrogen) and subcloned into pLitmus28i vector (HiScribeTM, New England Biolabs, Beverly, MA) containing two T7 promoters flanking the multi-cloning site. These promoters enabled amplification of the cloned fragment by using a T7 oligonucleotide. The PCR product was purified with QIAquick PCR purification kit (Qiagen, Valencia, CA). *In vitro* transcription of both DNA strands of the insert was performed with T7 RNA polymerase using the HiScribe RNAi Transcription Kit (New England Biolabs) as reported by the manufacturer, yielding dsRNA.

**Table 3:** Sequence of specific oligonucleotides used to amplify ATP synthase beta subunit, actin and heat shock protein genes.

Primer name	Oligonucleotide sequence	Product size
Ae-Act-F	5′ CCG GAA TTC CAA ACC AGC CAA AAT GTG TG	245 pb
Ae-Act-R	5′ CCC AAG CTT TTG GGT ACT TCA GGG TGA GG	
Ae-HeatShock-F	5′ CCG GAA TTC TTT CTC CCT GGA TGA ACC TG	227 pb
Ae-HeatShock-R	5′ CCC AAG CTT CGC TAG TGT GGG GAA GAG AG	
Ae-AtpS-F	5′ CCG GAA TTC GGA CAA GCT GAC CGT GGC CC	122 pb
Ae-AtpS-R	5′ CCC AAG CTT GAG GGA CCA GCT TTC CGG CG	
Ae-rps3-F	5′ GGA CGA AGC TCT TCT GGA TG	216 pb
Ae-rps3-R	5′ CCC ATT TGA TGA CAC AGT GC	

Silencing of specific proteins in *A. aegypti* was performed as previously described (8). Briefly, 200 neonates *A. aegypti* larvae were fed for 16 h with 200 µg of dsRNA previously encapsulated in Effectene transfection reagent (Qiagen, Valencia CA). For encapsulating the dsRNA in Effectene, 200 µg of dsRNA in a final volume of 4 ml of DNA-condensation buffer (EC buffer Qiagen), were mixed with 0.8 ml of Enhancer buffer (Qiagen) by vortexing and incubated 5 min at room temperature. Then the sample was mixed with 1.3 ml of Effectene by vortexing and incubated 10 min at room temperature. This sample was diluted in distilled water to a final volume of 10 ml where 200 larvae were added and incubated for 16 hours. After dsRNA feeding, the mosquito larvae were transferred to clean water and fed with regular diet (ground brewers yeast, lactalbumin and cat food Chow 1∶1∶1 ratio), until they reached fourth instar when bioassays were performed or guts were dissected for analysis by RT-PCR.

To confirm reduced transcript levels due to RNAi, we performed RT-PCR using 1 µg of total RNA isolated with RNeasy kit (Qiagen) and a First Strand cDNA Synthesis Kit (AMV, Roche). Using the specific primers reported in Table 3. Real time PCR or quantitative PCR (qRT-PCR) was performed to confirm proteomic studies. These experiments were performed with the ABI Prism7000 Sequence Detection System (Perkin-Elmer/Applied Biosystems) using the SYBR Green PCR Master Mix (Perkin-Elmer/Applied Biosystems). Experimental groups were the cDNA of three biological samples of midgut tissue isolated from 50 *A. aegypti* larvae treated with Cry11Aa toxin during 5 h with LC_50_, to analyze the regulation of *actin, hsp*90 and *ATP synthase* genes, and also the control reference gene that was *rps3* (AAEL008192). The control group was the cDNA of three biological samples of midgut tissue isolated from 50 *A. aegypti* larvae without toxin-intoxication, where same genes were analyzed. These pools of 50 midgut isolated from experimental or control larvae were submerged in 50 ml RNAlater stabilization reagent (Qiagen, Valencia, CA), and frozen separately at −80°C. The maximum storage time at −80°C was two weeks before processing. Total RNA was isolated from midgut tissue as described above. Primers used in qRT-PCR amplification were described in table 3. Oligo specificity to the target genes was assessed by Melt Curve and by BLAST search. The data were normalized using *rps3* gene as an internal control in quadruplicate rounds of the three independent biological samples. The quantification technique used to analyze data was the 2^−ΔΔ*C*^_T_ method [30]. Transcript levels with and without toxin were compared using ANOVA. Data plotted in figure 3 are expressed as relative transcription to time 0. All experiments were performed obtaining very similar values (differences of less than 0.3 SD). A non-template control reaction mixture was included for each gene. The specificity of the amplification products was confirmed by size estimations on a 2% agarose gel and by analyzing their melting curves. Each qRT-PCR reaction had a 12 µl reaction volume containing: cDNA corresponding to 5 ng input RNA, 1 µl of each forward primer and reverse primer (concentration of 10 pmol/µl), 6 µl of SYBR Green PCR Master Mix. The 96-Well Optical Reaction Plates with Barcode and ABI PRISM Optical Adhesive Cover lids were purchased from Applied Biosystems. Amplification conditions were: two min at 50°C for one cycle (stage one), 10 min at 95°C for one cycle (stage two); a two step cycle at 95°C for 15 s and 60°C for 60 s for a total of 40 cycles (stage three); and three step cycle at 95°C for 15 s and 60°C for 60 and 95°C for 15 s for one cycle (stage four- dissociation stage).
